# The effect of Fisher information matrix approximation methods in population optimal design calculations

**DOI:** 10.1007/s10928-016-9499-4

**Published:** 2016-11-01

**Authors:** Eric A. Strömberg, Joakim Nyberg, Andrew C. Hooker

**Affiliations:** Pharmacometrics Research Group, Department of Pharmaceutical Biosciences, Uppsala University, Box 471, 75124 Uppsala, Sweden

**Keywords:** Optimal design, Fisher information matrix, Full FIM, Block-diagonal FIM, FO, FOCE

## Abstract

With the increasing popularity of optimal design in drug development it is important to understand how the approximations and implementations of the Fisher information matrix (FIM) affect the resulting optimal designs. The aim of this work was to investigate the impact on design performance when using two common approximations to the population model and the full or block-diagonal FIM implementations for optimization of sampling points. Sampling schedules for two example experiments based on population models were optimized using the FO and FOCE approximations and the full and block-diagonal FIM implementations. The number of support points was compared between the designs for each example experiment. The performance of these designs based on simulation/estimations was investigated by computing bias of the parameters as well as through the use of an empirical D-criterion confidence interval. Simulations were performed when the design was computed with the true parameter values as well as with misspecified parameter values. The FOCE approximation and the Full FIM implementation yielded designs with more support points and less clustering of sample points than designs optimized with the FO approximation and the block-diagonal implementation. The D-criterion confidence intervals showed no performance differences between the full and block diagonal FIM optimal designs when assuming true parameter values. However, the FO approximated block-reduced FIM designs had higher bias than the other designs. When assuming parameter misspecification in the design evaluation, the FO Full FIM optimal design was superior to the FO block-diagonal FIM design in both of the examples.

## Introduction

Optimal design of clinical trials has become an increasingly popular and important tool in drug development to reduce the cost and increase informativeness of the study [1]. By utilizing a nonlinear mixed effects model (NLMEM) to describe the pharmacokinetic (PK) and pharmacodynamic (PD) properties of the drug, the Fisher information matrix (FIM) can be calculated for a set of design variables [2]. Through the Cramer-Rao inequality, the inverse of the FIM has been shown to give a lower bound of the Variance–Covariance matrix of model parameter estimates [3, 4]. By choosing design variables that maximize the FIM, the expected parameter uncertainty is minimized [5]. Different design criteria, which typically use the FIM in the calculation of a single-valued objective function may be used to compare and optimize the designs [6]. One of the simplest and most common design criteria is called D-Optimality which compares designs using the determinant of the FIM under the assumption that all estimated parameters are of equal importance.

The computation of the FIM is of high numerical complexity and, with the lack of an analytical expression for the likelihood in NLMEMs, the exact solution to the FIM cannot be derived. Therefore, several approximations of the FIM are available which can yield slightly different results and affect the resulting optimal designs. Typically, first order approximations to the NLMEM are used in computation of the FIM, which linearize the random effects of the NLMEM around a mean of 0 (FO) or around individual realizations of the random effects (FOCE) [7]. Furthermore, there are two implementations of the FIM that are commonly used today; the full FIM and a block-diagonal FIM, which is formed under the assumption that the random effects components of an NLMEM are independent of the typical values [8–10]. Previous work in evaluating a specific design has shown that similar results are achieved when using the same approximation in different optimal design software (PFIM, PopED, PopDes, POPT and PkStaMP) [11]. It has also been shown that in some cases the block-diagonal FIM is a better predictor than the full FIM of the results gained from a design when evaluated using Monte Carlo based methods [10, 11]. However, other work has shown that the full FIM is the superior implementation [9]. Little work has been done to compare these methods when using them in the optimization of experimental designs (not just evaluation).

One common way to compare designs is through a FIM evaluation, where a lower bound of the expected parameter uncertainty can be assessed [11]. However, when comparing different approximations and implementations of the FIM, this approach will not work, since the resulting FIM will depend on the approximation used in the evaluation. To objectively compare the different designs, this work employs a Monte Carlo simulation/estimation based procedure to generate the empirical variance–covariance matrix (empCOV) and transform it to an empirical D-criterion. There is however uncertainty in this calculation of the empirical variance–covariance matrix, dependent on the number of simulations and which has to be taken into consideration when comparing designs. This work proposes that this uncertainty can be addressed by computing confidence intervals of the empirical D-criterion using bootstrap methodology.

For optimizations of sampling designs where there are more samples per individual than model parameters, the resulting optimal designs have been shown to include clustering of samples on model parameter dependent support points [12]. The effect of this clustering of samples may be beneficial if the model is true (in terms of structure AND parameter values) since it adds additional information for the parameters in the true model. However, if, in a design calculation, the assumptions of model structure or model parameter values are incorrect, then clustering of samples will potentially occur at non-optimal points.

In this work the effect of using the approximation methods FO and FOCE and the full and block-diagonal FIM on D-optimal designs is investigated for two commonly used NLMEMs. The number of support points (and thus the amount of clustering) for each FIM implementation and approximation method is compared and the effect of parameter misspecification in the design stage on the design performance is evaluated. It is hypothesized a D-optimal design with more support points and less clustering will be more robust to parameter misspecification in the design calculations.

## Background theory

### Evaluation of the Fisher information matrix

The individual response, $y_{i}$, given the individual design vector, *ξ*_*i*_, can, for a NLMEM, be written as

$$y_{i} = {\text{f}}\left( {\theta_{i} , \xi_{i} } \right) + {\text{h}}\left( {\theta_{i} , \xi_{i} , \varepsilon_{i} } \right)$$

where f(.) is the structural model, h(.) is the residual error model and $\varepsilon_{i}$ is the residual error vector. The individual parameters are given by the vector $\theta_{i} = {\text{g}}\left( {\beta_{ } , \eta_{i} } \right)$ which is dependent on the fixed effects vector $\beta$, containing the typical values of the parameters, and the vector of random effects $\eta_{i}$ which describes the subject specific deviations from the typical values. In this work, the *j* between subject variability (BSV) terms and the *k* residual unexplained variability (RUV) terms are assumed to be normally distributed with mean zero and respective covariance matrices **Ω** and ${\varvec{\Sigma}}$ of size (*k*, *k*) and (*j*, *j*).

The Fisher information matrix for the *i*th individual with the vector of design variables *ξ*_*i*_ and expected response E(*y*_*i*_) and variance V(*y*_*i*_)can be written as [12]

$${\text{FIM}}_{i}^{FULL} \left( {\Theta ,\xi_{i} } \right) = \frac{1}{2}\left[ {\begin{array}{*{20}c} {\varvec{A}\left( {{\text{E}}\left( {y_{i} } \right) ,{\text{V}}\left( {y_{i} } \right)} \right)} & {\varvec{C}\left( {{\text{V}}\left( {y_{i} } \right)} \right)} \\ {\varvec{C}\left( {{\text{V}}\left( {y_{i} } \right)} \right)} & {\varvec{B}\left( {{\text{V}}\left( {y_{i} } \right)} \right)} \\ \end{array} } \right]$$

$$\varvec{A}\left( {{\text{E}}\left( {y_{i} } \right) ,{\text{V}}\left( {y_{i} } \right))} \right) = 2 \cdot \frac{{\partial {\text{E}}\left( {y_{i} } \right)}}{\partial \beta }^{T} {\text{V}}\left( {y_{i} } \right)^{ - 1} \cdot \frac{{\partial {\text{E}}\left( {y_{i} } \right)}}{\partial \beta } + tr\left( {\frac{{\partial {\text{V}}\left( {y_{i} } \right)}}{\partial \beta } \cdot {\text{V}}\left( {y_{i} } \right)^{ - 1} \cdot \frac{{\partial {\text{V}}\left( {y_{i} } \right)}}{\partial \beta } \cdot {\text{V}}\left( {y_{i} } \right)^{ - 1} } \right)$$

$$\varvec{B}\left( {{\text{V}}\left( {y_{i} } \right)} \right) = tr\left( {\frac{{\partial {\text{V}}\left( {y_{i} } \right) }}{\partial \lambda } \cdot {\text{V}}\left( {y_{i} } \right)^{ - 1} \cdot \frac{{\partial {\text{V}}\left( {y_{i} } \right) }}{\partial \lambda } \cdot {\text{V}}\left( {y_{i} } \right)^{ - 1} } \right)$$

$$\varvec{C}\left( {{\text{V}}\left( {y_{i} } \right)} \right) = tr\left( {\frac{{\partial {\text{V}}\left( {y_{i} } \right) }}{\partial \beta } \cdot {\text{V}}\left( {y_{i} } \right)^{ - 1} \cdot \frac{{\partial {\text{V}}\left( {y_{i} } \right) }}{\partial \beta } \cdot {\text{V}}\left( {y_{i} } \right)^{ - 1} } \right)$$

where Θ = [*β*, *λ*] = [*β*, ω_1_^2^, …, ω_*k*_^2^, σ_1_^2^, …, σ_*j*_^2^] is the vector of population parameters containing the fixed effects parameters *β* and the variance/covariance terms in the Ω and Σ matricies. By assuming that the variance of the model ${\text{V}}\left( {y_{i} } \right)$ is independent of the change in typical values $\beta$, $\varvec{C}\left( {{\text{V}}\left( {y_{i} } \right)} \right)$ is zero and the Full FIM can be reduced to its block-diagonal form [13]:

$${\text{FIM}}_{i}^{\text{block-diag}} \left( {\Theta ,\xi_{i} } \right) = \frac{1}{2}\left[ {\begin{array}{*{20}c} {\varvec{A}\left( {{\text{E}}\left( {y_{i} } \right),{\text{V}}\left( {y_{i} } \right)} \right)} & 0 \\ 0 & {\varvec{B}\left( {{\text{E}}\left( {y_{i} } \right),{\text{V}}\left( {y_{i} } \right)} \right)} \\ \end{array} } \right]$$

with the A block being reduced to

$$\varvec{A}\left( {{\text{E}}\left( {y_{i} } \right) ,{\text{V}}\left( {y_{i} } \right)} \right) = 2 \cdot \frac{{\partial {\text{E}}\left( {y_{i} } \right)}}{\partial \beta }^{T} {\text{Var}}\left( {y_{i} } \right)^{ - 1} \cdot \frac{{\partial {\text{E}}\left( {y_{i} } \right)}}{\partial \beta }$$

The expected model response ${\text{E}}\left( {y_{i} } \right)$ and variance ${\text{V}}\left( {y_{i} } \right)$ are approximated by linearizing the NLME model with respect to the BSV, *η*_*i*_, and the RUV, *ɛ*_*i*_, to guarantee marginal normally distributed observations. One of the two most common linearizations, which have been used in this work, is the First Order (FO) linearization, which linearizes each individual’s random effects around the typical values $\theta_{i,0} = g\left( {\beta_{ } ,\eta_{i} = 0} \right)$ and *ɛ*_*i*_ = 0. This gives the individual response function for a single occasion

$$y_{i} \approx {\text{f}}\left( {\theta_{i,0} , \xi_{i} } \right) + \eta_{i} \cdot {\mathbf{L}}_{i} \left( {\theta_{i,0} , \xi_{i} } \right) + \varepsilon_{i} \cdot {\mathbf{H}}_{i} \left( {\theta_{i,0} , \xi_{i} ,\varepsilon_{i} = 0} \right)$$

where ${\mathbf{L}}_{i} \left( {\theta_{i,0} , \xi_{i} } \right) \equiv \frac{{\partial {\text{f}}}}{\partial \eta }\left( {\theta_{i,0} , \xi_{i} } \right)$ and ${\mathbf{H}}_{i} \left( {\theta_{i,0} , \xi_{i} ,\varepsilon_{i} = 0} \right) \equiv \frac{{\partial {\text{h}}}}{\partial \varepsilon }\left( {\theta_{i,0} , \xi_{i} ,\varepsilon_{i} = 0} \right)$. Since *η*_*i*_ = 0 and *ɛ*_*i*_ = 0 this gives the expected response and variance

$${\text{E}}_{FO} \left( {y_{i} } \right) \approx {\text{f}}\left( {\theta_{i,0} , \xi_{i} } \right)$$

$${\text{Var}}_{FO} \left( {y_{i} } \right) \approx {\mathbf{L}}_{i} \left( {\theta_{i,0} , \xi_{i} } \right) \cdot {\varvec{\Omega}} \cdot {\mathbf{L}}_{i}^{{\mathbf{T}}} \left( {\theta_{i,0} , \xi_{i} } \right) + diag\left( {{\mathbf{H}}_{i} \left( {\theta_{i,0} , \xi_{i} } \right) \cdot {\varvec{\Sigma}} \cdot {\mathbf{H}}_{i}^{{\mathbf{T}}} \left( {\theta_{i,0} , \xi_{i} } \right)} \right)$$

This approximation can, however, be inaccurate when the BSV becomes large, is highly nonlinear or when the interaction of the residual error and inter-individual random effect is important. A better, but more time-consuming approximation is the first order conditional estimate (FOCE) linearization; this method linearizes the expectation of the model $E_{ } \left( {y_{i} } \right)$ around individual samples of the BSV taken from a normal distribution of $\tilde{\eta }_{i} \sim N\left( {0,\Omega } \right)$ [14]. This gives the expected response and variance for a single occasion

$$E_{FOCE} \left( {y_{i}}\right) \approx \left({\tilde{\theta }_{i}, \xi_{i}} \right) - \tilde{\eta }_{i}^{{\mathbf{T}}} \cdot {\mathbf{L}}_{i} \left( {\tilde{\theta }_{i} , \xi_{i} } \right)$$

$${\text{Var}}_{FOCE} \left( {y_{i} } \right) \approx {\mathbf{L}}_{i} \left( {\tilde{\theta }_{i} , \xi_{i} } \right) \cdot {\varvec{\Omega}} \cdot {\mathbf{L}}_{i}^{{\mathbf{T}}} \left( {\tilde{\theta }_{i} , \xi_{i} } \right) + diag\left( {{\mathbf{H}}_{i} \left( {\tilde{\theta }_{i} , \xi_{i} } \right) \cdot {\varvec{\Sigma}} \cdot {\mathbf{H}}_{i}^{{\mathbf{T}}} \left( {\tilde{\theta }_{i} , \xi_{i} } \right)} \right)$$

where $\tilde{\theta }_{i} = g\left( {\beta ,\tilde{\eta }_{i} } \right)$. A more detailed description of different linearizations and the derivation of the FIM is reported in Nyberg et al. and Retout and Mentré [12, 14].

## Methods

Two representative pharmacometric models and their initial designs were optimized using the FO or FOCE model linearization and the full FIM calculation or the block-diagonal FIM approximation. Evaluation of these four different designs per example were then performed using stochastic simulation and estimation assuming either that the optimal designs were computed with the correct model parameter values or misspecified model parameter values.

### Example 1: warfarin PK

The Warfarin PK model and design was previously utilized by Nyberg et al. [11] and Bazzoli et al. [15] in optimal design evaluations. The model is one compartment with linear absorption, log-normal BSV on all parameters and an additive and proportional RUV. That is, for the *ith* individual with response *y*_i_, the set of individual parameters *θ*_*i*_ and individual sampling times vector *t*_*i*_ we have:$y_{i} \left( {t_{i} ,\theta_{i} } \right) = \frac{{Dose k_{a,i} \frac{{Cl_{i} }}{{V_{i} }}}}{{\left( {Cl_{i} \left( {k_{a,i} - \frac{{Cl_{i} }}{{V_{i} }}} \right)} \right)}}\left( {e^{{ - \frac{{Cl_{i} }}{{V_{i} }} t_{i} }} - e^{{ - k_{a,i} t_{i} }} } \right)\left( {1 + \varepsilon_{prop,i} } \right) + \varepsilon_{add,i} \left( {{\text{mg}}/{\text{L}}} \right)$

$$Cl_{i} = \beta_{CL} \cdot e^{{\eta_{CL,i} }} \left( {{\text{L}}/{\text{h}}} \right)$$

$$V_{i} = \beta_{V} \cdot e^{{\eta_{V,i} }} \left( {\text{L}} \right)$$

$$k_{a,i} = \beta_{Ka} \cdot e^{{\eta_{Ka,i} }} ({\text{h}}^{ - 1} )$$

The initial study design from Nyberg et al. consisted of 32 individuals in one group after a single fixed dose of 70 mg and sampling schedule $t_{init} = \left( {0.5, 2, 3, 6, 24, 36, 72, 120} \right)$ hours. All model parameters are listed in Table 1.

**Table 1:** Parameter values of the warfarin example model

Fixed effects		Random effects	
$\varvec{\beta}_{{\varvec{CL}}}$ (L/h)	0.15	$\varvec{\omega}_{{\varvec{ Cl}}}^{2}$	0.07
$\varvec{\beta}_{\varvec{V}}$ (L)	8	$\varvec{\omega}_{{\varvec{V }}}^{2}$	0.02
$\varvec{\beta}_{{\varvec{Ka}}}$ (h^−1^)	1	$\varvec{\omega}_{{\varvec{Ka }}}^{2}$	0.6
Covariates		$\varvec{ \sigma }_{{\varvec{ prop}}}^{2}$	0.1
Dose (mg)	70 FIX	$\varvec{\sigma}_{{\varvec{ add }}}^{2} \varvec{ }\left[ {{\mathbf{mg}}/{\mathbf{L}}} \right]$	0.01 FIX

The Warfarin model used by Nyberg et al. has only a proportional residual variability. The model used here has an additional fixed additive component representing an assumed, known, assay error. This extra error term will help avoid optimal samples at very low concentrations that would be practically below the quantification limit [16].

### Example 2: PD—sigmoidal EMAX model

The second example is a sigmoidal EMAX model with the dose of a drug as the independent variable, implemented as:

$$y_{i} \left( {D_{i} ,\theta_{i} } \right) = \frac{{D_{i}^{\gamma } Emax_{i} }}{{\left( {ED50_{i}^{\gamma } + D_{i}^{\gamma } } \right)}}\left( {1 + \varepsilon_{prop,i} } \right) + \varepsilon_{add,i}$$

$$Emax_{i} = \beta_{Emax} \cdot e^{{\eta_{Emax,i} }}$$

$$ED50_{i} = \beta_{ED50} \cdot e^{{\eta_{ED50,i} }}$$

$$\gamma_{i} = \beta_{\gamma } \cdot e^{{\eta_{\gamma ,i} }}$$

A combined additive and proportional residual variability was chosen so that the total maximum magnitude of residual variability was 10% of the maximum model response. The initial design was 100 individuals in one group with 8 sampling events between 0 and 50 dose units (d.u), and the initial sampling schedule $D_{init} = \left( {1, \, 5, \, 10, \, 15, \, 20, \, 30, \, 40, \, 50} \right)$ d.u. All the model parameter values can be found in Table 2. As in example 1, a fixed additive error was used to avoid optimal doses that would lead to effect measurements below the quantitative limit.

**Table 2:** Parameter values of the EMAX example model

Fixed effects		Random effects	
$\varvec{\beta}_{{\varvec{EMAX}}}$	100	$\varvec{\omega}_{{\varvec{EMAX}}}^{2}$	0.0625
$\varvec{\beta}_{{\varvec{ED}50}}$	20	$\varvec{\omega}_{{\varvec{ED}50\varvec{ }}}^{2}$	0.0625
$\varvec{\beta}_{\varvec{\gamma}}$	4.5	$\varvec{\omega}_{{\varvec{\gamma }}}^{2}$	0.0625
		$\varvec{\sigma}_{{\varvec{ prop}}}^{2}$	0.0025
		$\varvec{\sigma}_{{\varvec{ add}}}^{2}$	25 FIX

### Design optimization

The sampling schedule for both example models, was optimized using D-optimality in PopED 2.13 for MATLAB [12, 17]. Four optimal designs were generated per example by using the FO and FOCE model linearizations to approximate the full and the block-diagonal FIM. The default optimization settings were used and the FOCE approximation had 4000 conditional samples. The FO optimizations using the full and block-diagonal FIM were replicated in PFIM 3.2.2 [15] with the default optimization search settings for comparison of the optimal designs generated with PopED.

### Design evaluation without parameter misspecification

**Fig. 1:**
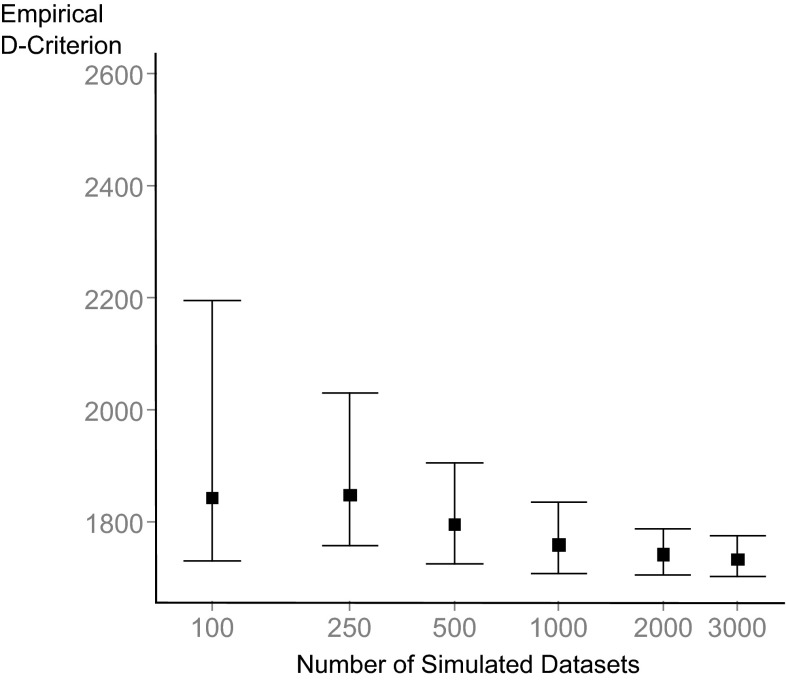
95% confidence intervals of the empirical D-criterion for the initial design in the Warfarin example as the number of simulated datasets and estimated parameter vectors increase. The confidence intervals are generated by a 1000 iteration bootstrap of COV-matrix calculations from the SSE parameter vectors based on 100–3000 simulated datasets. The *lower and upper lines* represent the 5th and 95th percentiles respectively. The *dot* represents the empirical D-criterion calculated without bootstrap

To evaluate the different designs, stochastic simulation and estimations (SSE) were run using Pearl speaks NONMEM (PsN) [18]. The example models were used to simulate 3000 datasets for each optimized design using the same parameters used in the design optimization (Tables 1, 2). The model parameters were then re-estimated to fit the simulated data (All parameter estimation was done using the FOCEI approximation in NONMEM 7.3 [19]), which generated 3000 estimated parameter vectors per SSE. These parameter vectors were then used to derive an empirical variance–covariance matrix (COV) and the empirical D-criterion:

$$D - Criterion_{Emp.} = \left( {\frac{1}{\text{COV}}} \right)^{1/p}$$

where p is the number of estimated parameters. However, as demonstrated in Fig. 1, the calculation of the empirical D-criterion from these parameter vectors is dependent on the number of simulations and estimated parameter vectors included in the calculation. This variability, caused by the nature of Monte-Carlo simulations, makes it possible for two SSEs for the same design and model to give different empirical D-criterion. This could in practice lead to false conclusions when comparing the empirical D-criteria of two candidate designs. To find the “true” Empirical D-criterion the number of simulated datasets would have to approach infinity. To counteract the risk of false conclusions, 95% confidence intervals of the D-criterion were generated using a case-resampling bootstrap with 10,000 iterations [20].

In addition, for each SSE, the absolute relative bias and relative estimation error (REE) for each parameter was calculated:

$$relative\; bias \left( {\Theta_{i} } \right) = abs\left( {\frac{1}{N}\left( {\mathop \sum \limits_{j}^{N} \frac{{\hat{\Theta }_{i,j} - \Theta_{i} )}}{{\Theta_{i} }}} \right)} \right)$$

$$REE\left( {\Theta_{i} } \right) = \frac{{\hat{\Theta }_{i,j} - \Theta_{i} }}{{\Theta_{i} }}$$

where $\hat{\Theta }_{i,j}$ is the jth estimate of parameter i, from N re-estimations of the true parameter Θ_*i*_ used in the dataset simulation.

### Design evaluation with parameter misspecification

Design robustness was evaluated by emulating parameter misspecification in the design stage. The example models were used to simulate 3000 datasets for each optimized design using a range of parameter vectors that were different than those used in the design optimization (design calculations were always based on the parameters in Tables 1, 2). The simulation parameters were generated by randomly perturbing all model parameters using a uniform distribution of 50–200% of the value used in the design optimization. The model parameters were then re-estimated to fit the simulated data.

Thus, for each SSE in this design evaluation with parameter misspecification, the true parameter values used for simulation were different for each parameter vector. This increased the variability between the parameter vectors, which would inflate the empirical variance–covariance matrix (COV) and the empirical D-criterion. Therefore, the following correction was applied to the re-estimated parameters, when computing the empirical D-criterion intervals:

$$\hat{\Theta }_{i,j} = \hat{\Theta }_{SSE,j} + \left( {\Theta - \Theta_{SSE,j} } \right)$$

where $\hat{\Theta }_{c,j}$ is the *jth* corrected parameter vector,$\hat{\Theta }_{SSE,j}$ is the re-estimated parameter vector based on simulation *j*,$\Theta$ is the parameter vector used for design optimization and Θ_*SSE*,*j*_ is the perturbed and true parameter vector that was used for simulation of dataset *j*. The absolute relative bias and REE were computed as in the previous section with the uncorrected parameter values.

## Results

For both investigated models, the optimizations using the full FIM implementation increased the number of support points and reduced sample clustering when compared to the block-diagonal FIM implementations. Further, the optimizations using the FOCE approximation increased the number of support points and reduced sample clustering when compared to the FO approximation.

For the Warfarin model, when using the FO approximation and the full FIM for optimization, the optimal sampling schedule was shown to have 5 support points with the optimal sampling schedule $t_{full}^{FO} = \left( {0.14, \, 2.18, \, 2.18, \, 2.18, \, 8.97, \, 8.97, \, 53.1, \, 120} \right).$Optimizations using the block-diagonal FIM yielded 3 support points and the sampling schedule $t_{\text{block-diag.}}^{FO} = \left( {0.26, \, 6.33, \, 6.33, \, 6.33, \, 6.33, \, 6.33, \, 6.33, \, 120} \right).$Optimizing using the FOCE approximation and the full FIM gave designs with 8 support points and the optimal sampling schedule $t_{Full}^{FOCE} = \left( {0.05, \, 0.36, \, 1.13, \, 4.65, \, 6.73, \, 19.6, \, 41.7, \, 120} \right).$Using the block-diagonal FOCE FIM yielded 7 support points and the optimal sampling schedule $t_{\text{block-diag.}}^{FOCE} = \left( {0.04, \, 0.29, \, 0.70, \, 5.73, \, 5.74, \, 13.7, \, 27.8, \, 120} \right).$

For the EMAX example, the optimal sampling schedules optimized using the FO approximation were $D_{Full}^{FO} = \left( {12.8, \, 12.8, \, 19.4, \, 28.0, \, 28.0, \, 50.0, \, 50.0, \, 50.0} \right)$ with 4 support points for the full FIM and $D_{\text{block-diag.}}^{FO} = \left( {13.8, \, 13.8, \, 25.4, \, 25.4, \, 50.0, \, 50.0, \, 50.0, \, 50.0} \right)$ with 3 support points for the block diagonal FIM. When using the FOCE approximation the optimal sampling schedules were $D_{Full}^{FOCE} = \left( {11.6, 15.4, 19.9, 24.6, 30.0, 37.2, 50.0, 50.0} \right)$ and $D_{\text{block-diag.}}^{FOCE} = \left( {12.2, \, 16.4, \, 21.4, \, 26.5, \, 35.3, \, 50, \, 50.0, \, 50.0} \right)$ with 7 and 6 support points for the full and block-diagonal FIMs respectively.

The optimizations performed with PopED agreed well with the results from PFIM and gave final designs that were identical to the third significant digit when optimizing with the block diagonal and full FO FIM.

**Fig. 2:**
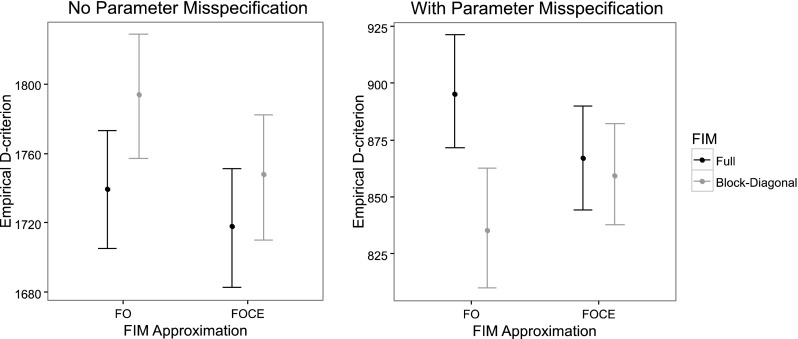
Comparison of empirical D-criterion for the 8-point design in the warfarin example, optimized using the FO and FOCE algorithms and using the full and block diagonal FIMs. The median and the 5th and 95th percentiles of the empirical D-criterions were calculated from a bootstrap of estimated parameter vectors from an SSE with 3000 datasets with (*left panel*) and without (*right panel*) misspecification in the design calculations. The *dot* represents the bootstrap median while the *top and bottom lines* represents the 95th and 5th percentiles respectively

**Fig. 3:**
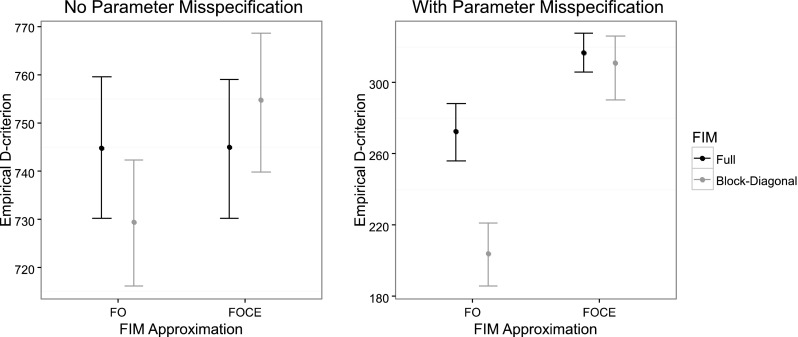
Comparison of Empirical D-criterion for the 8-point design in the EMAX example, optimized using the FO and FOCE algorithms and using the full and block-diagonal FIMs. The median and the 5th and 95th percentiles of the empirical D-criterions were calculated from a bootstrap of estimated parameter vectors from an SSE with 3000 datasets with (*left panel*) and without (*right panel*) misspecification in the design calculations. The *dot* represents the bootstrap median while the *top and bottom lines* represents the 95th and 5th percentiles respectively

**Fig. 4:**
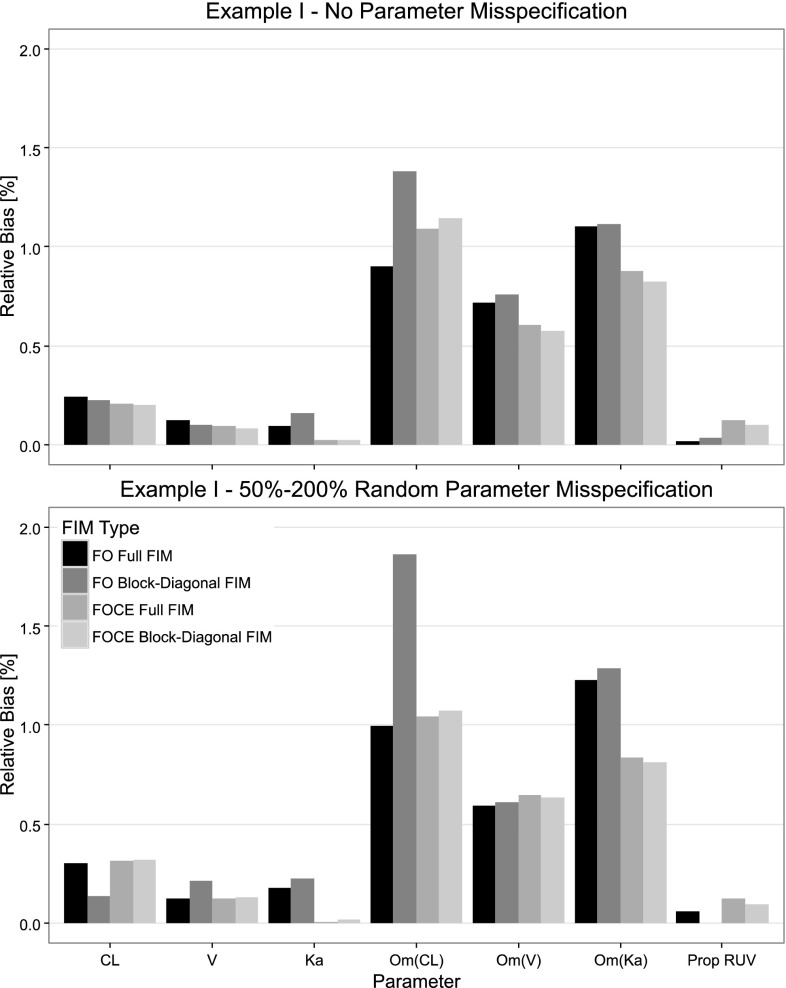
The absolute mean relative bias for the warfarin model parameters for four different designs optimized using the FO and FOCE approximations and the full and block-diagonal FIMs. The designs were evaluated using SSEs with 3000 simulated datasets where parameter misspecification was included for the *bottom panel*

**Fig. 5:**
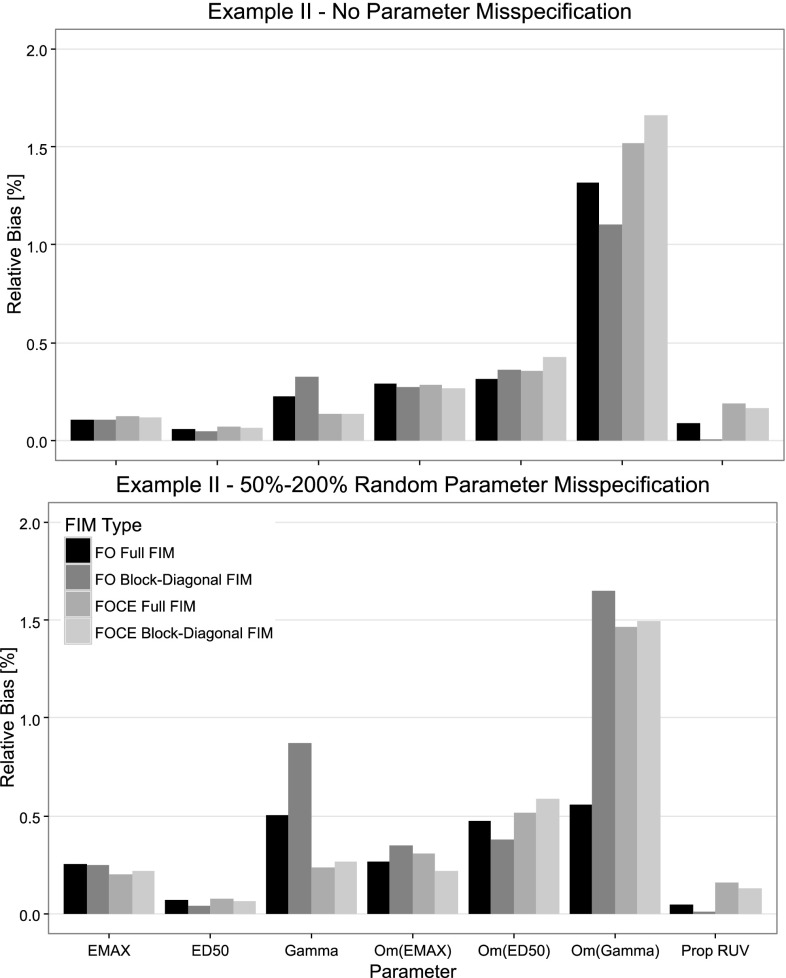
The absolute mean relative bias for the EMAX model parameters for four different designs optimized using the FO and FOCE approximations and the Full and Block-Diagonal FIMs. The designs were evaluated using SSEs with 3000 simulated datasets where parameter misspecification in the design calculation was included for the *bottom panel*

When evaluating the D-optimal designs using SSEs and assuming no parameter misspecification in the design phase, no significant differences in D-criterion between the optimal designs of the full and block-diagonal FIM implementations was observed for these examples. Furthermore, there was no significant advantage in the FOCE designs over the FO designs according to the empirical D-criterion (left plot in Figs. 2, 3). The FO block-diagonal design had, however, a slightly higher D-criterion than the FOCE Full FIM design in the warfarin example. In both examples, the design using the block-diagonal FIM and the FO approximation resulted in a slightly higher bias for the fixed effect and BSV parameter estimates (top plot in Figs. 4, 5) compared to the Full FIM designs. These differences were less noticeable when the FOCE approximation was used, which for some parameters increased the bias.

With Parameter misspecification included in the evaluation of the D-optimal designs, the FO designs based on the full FIM were superior (higher D-criterion, more information, less parameter variability) to the FO block diagonal designs in both examples (right plot in Figs. 2, 3). For the EMAX example, the FOCE optimal designs both resulted in a higher D-criterion than the FO designs. Further, in both examples, the design based on the block-diagonal FIM using the FO approximation resulted in the parameter with the largest relative bias (bottom plot in Figs. 4, 5). The bias of parameter estimates was reduced for the block-diagonal FIM design when the FOCE approximation was used.

**Fig. 6:**
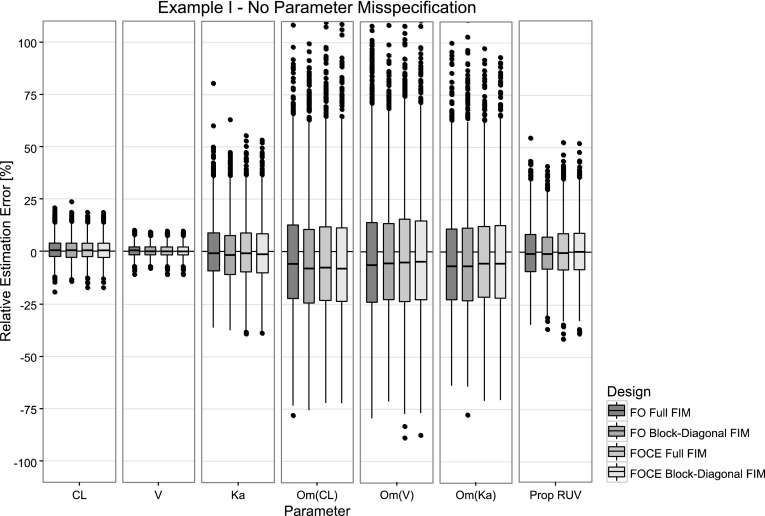
Standard boxplots of relative estimation error of the Warfarin model parameters for four different designs optimized using the FO and FOCE approximations and the full and block-diagonal FIM implementations. The estimation error was calculated from parameter vectors generated by stochastic simulations and estimations with 3000 simulated datasets

**Fig. 7:**
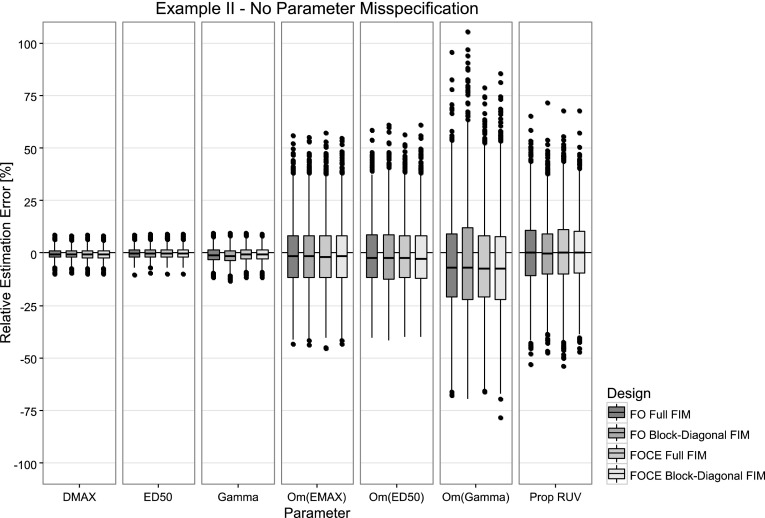
Standard boxplots of relative estimation error of the EMAX model parameters for four different designs optimized using the FO and FOCE approximations and the full and block-diagonal FIM implementations. The estimation error was calculated from parameter vectors generated by stochastic simulations and estimations with 3000 simulated datasets

**Fig. 8:**
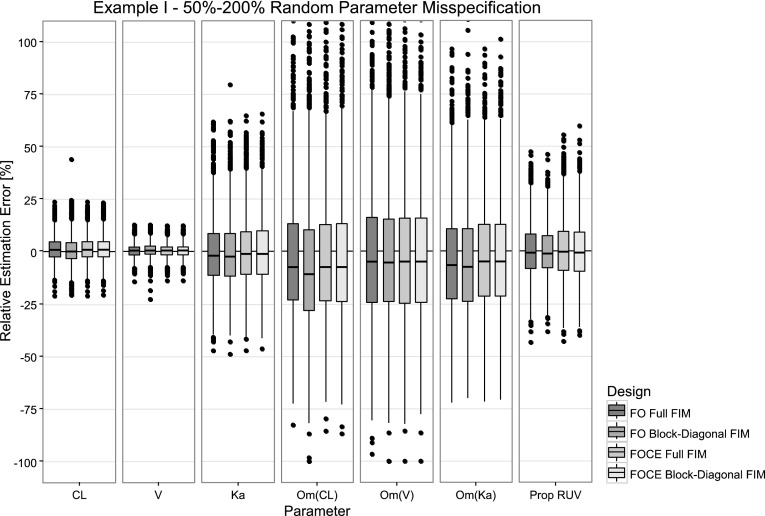
Standard boxplots of relative estimation error of the Warfarin model parameters, with parameter misspecification in the design stage, for four different designs optimized using the FO and FOCE approximations and the full and block-diagonal FIM implementations. The estimation error was calculated from parameter vectors generated by stochastic simulations and estimations with 3000 simulated datasets. The parameters used for dataset simulation were randomly perturbed to 50–200% of the value used in the design optimization to emulate parameter misspecification in the design stage

**Fig. 9:**
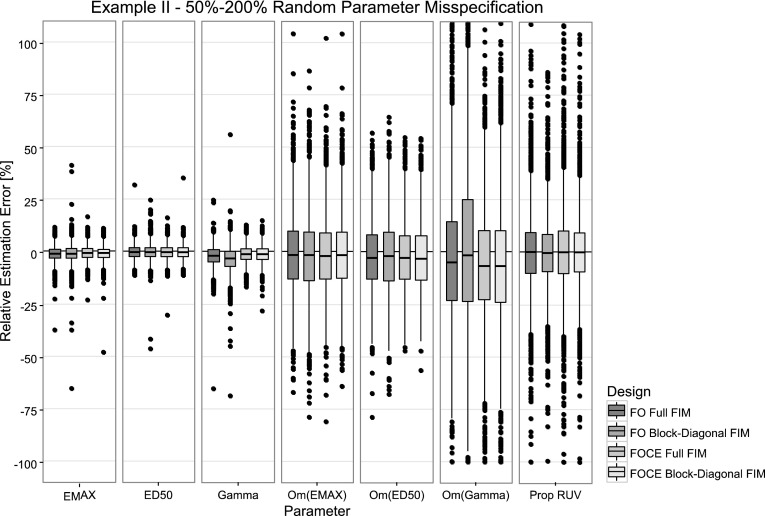
Standard boxplots of relative estimation error of the EMAX model parameters, with parameter misspecification in the design stage, for four different designs optimized using the FO and FOCE approximations and the full and block-diagonal FIM implementations. The estimation error was calculated from parameter vectors generated by stochastic simulations and estimations with 3000 simulated datasets. The parameters used for dataset simulation were randomly perturbed to 50–200% of the value used in the design optimization to represent parameter misspecification in the design stage

Boxplots of relative estimation error for the parameters are available as supplementary figures (Figs. 6, 7, 8, 9) in the Appendix.

## Discussion

This work compares how the full and block-diagonal Fisher information matrices and two common FIM linearizations affect the optimal design when optimizing sampling schedules for two different example models. These examples reveal that optimizing with the full FIM and the FOCE approximation could increase the number of support points and yield reduced clustering in the optimal designs compared to designs optimized with the FO approximation and block-diagonal FIM. The designs effect on estimated parameter accuracy and precision were evaluated by stochastic simulations and re-estimations, which is generally considered the gold standard in design evaluation. When model parameters were correctly specified in the example design calculations, then all designs generally performed equally well. When model parameters were misspecified in the example design calculations, the designs from the most commonly used FO, block-diagonal FIM (the designs with the lowest number of support points) perform worse than the designs from the FO, full FIM designs. Use of the, considerably more time consuming, FOCE approximation was beneficial in one of the examples but not the other.

For models without between-subject variability, where the FIM has an analytic solution, the expected number of optimal design support points should be equal to the number of estimated fixed effects parameters [21]. For nonlinear mixed effects models, the number of support points can depend on the underlying model structure as well as parameters [22], but it may be reasonable to assume that the number of support points should be between the number of fixed effect parameters and the total number of fixed and random effect parameters. For all examples in this work, the number of support points was never less than the number of fixed effect parameters. However, the number of support points did exceed the total number of estimated parameters (both fixed and random) in the FOCE, full FIM design for example 1 with 8 support points for 7 estimated parameters. To test that this design was not a local maximum, the 8-point design was manually validated by iteratively reducing the final design by moving all samples on one support point to another support point and then re-running the optimizations. The optimization algorithm converged upon the same 8-point design regardless of initial design.

In this work we see that the number of support points in a design will depend on the approximations used in the FIM calculation. For both examples, the designs optimized using the FO approximated FIMs had fewer support points and more clustered samples than the designs based on the FOCE FIMs. In addition, using the Full FIM in the optimization resulted in more support points and less clustering compared to when the block-diagonal FIM was utilized. These results may be explained by examining the approximations used in each calculation. When optimizing the designs using the FO approximation, the individual response is linearized around the mean of the random effects, which may be reflected in the lower number of support points since the information regarding the BSV is for this linearization is gained at the sampling points where the most information regarding the fixed effect parameters are found. By using the FOCE approximation, additional information regarding the BSV parameters are required since FOCE linearizes around individual samples of the response. This may increase the number of support points in the FOCE designs relative to the FO designs, and may increase the support points beyond the number needed for population parameter estimation in favor of individual parameter estimation. Additionally, when BSV and RUV parameters are included in the model such that the variance of the model is dependent on fixed effect parameters (for example, with log-normal BSV parameters or proportional RUV parameters) then the block-diagonal FIM will ignore this information potentially resulting in fewer support points compared to the full FIM.

When the designs were evaluated via SSE experiments without parameter misspecification in the design calculations, there were no differences in D-criterion between the full and block-diagonal FIM designs. In addition, there were no differences in D-criterion between the FO and FOCE designs, given the same matrix block assumptions. However, when the designs were evaluated via SSE experiments with parameter misspecification in the design calculations, the FO Full FIM design was superior to the FO block-diagonal design in both examples. In addition, the FO block-diagonal design in the warfarin example was no longer superior to the FOCE full FIM design, and the FOCE designs were superior to the FO block-diagonal design in the EMAX example. This could be due to the higher number of support points in the Full FIM and FOCE designs which made the designs less sensitive to parameter misspecification in the design stage. Additionally, the parameter with highest bias occurred, in both examples, for the FO block-diagonal design when the designs were optimized with parameter misspecification.

Clearly, the clustering of samples at support points is also of practical concern. It requires multiple samples taken simultaneously which is not always possible. This work indicates that the full FIM approximation may increase the number of support points in a D-optimal design. There are other methods that could be used in combination with the full FIM to avoid clustering of samples. One could apply sampling windows around the optimal sampling times in which samples may be taken at an accepted loss of design efficiency [23]. A second approach is to reduce the clustering is to include information about residual error autocorrelation in the model [24, 25]. A third approach is to incorporate information about parameter uncertainty in the design calculation using E-family design calculations, which have been shown to be more robust to parameter misspecification in the design stage [26].

It should be noted that the FOCE approximation used in these examples differs from the implementation of FOCE used by NONMEM where the model is linearized around the mode of the joint density function for each subject based on the observed data for each individual. In this work we assume that the model is linearized around normally-distributed individual parameter estimates assuming these estimates come from the population distribution without shrinkage. An updated FOCE approximation, FOCE_Mode_, which linearizes around the Empirical Bayes Estimate (EBE) for each individual could be used [12]. However, in this work, the design was rich, thus the shrinkage of individual parameter estimates should be minimal and the approximations should be similar.

Finally, when comparing designs for models with many parameters, it can sometimes be difficult to determine the overall best design. The empirical D-criterion from stochastic simulation and estimation facilitated a quick overview of design performance that was independent of the approximation method and FIM implementation used in the design stage. Despite the high number of simulations and estimations, there was still uncertainty in the calculation of this empirical D-criterion due to the simulation and estimation approach. Therefore, comparing the designs based on a single estimate of the empirical D-criterion would have led to false conclusions regarding differences in design performance. For instance, *t*_*block*-*diag*._^*FO*^ would have seemed superior to *t*_*Full*_^*FO*^ when the designs were calculated without model parameter misspecification. By comparing 95% confidence intervals of the empirical D-criterion, false differences between designs caused by this uncertainty were reduced, and actual differences could be observed at a 95% level of confidence. To fully evaluate the designs however, standard metrics such as parameter bias and standard errors are still recommended to use as a complement to the empirical D-criterion intervals. In particular, for scenarios where certain parameters in the model are the main interest.

## Conclusions

Using the full rather than the block-diagonal FIM and FOCE instead of FO for design optimization could increase the number of support points and reduce clustering of sampling times in a design. The Full FIM can increase the design robustness and thus be less sensitive to parameter misspecification in the design stage compared to block-diagonal FIM designs.

Confidence intervals of the D-criterion reduce the risk of false conclusions caused by the uncertainty in the variance–covariance matrix calculations, and may be useful as an initial design diagnostic and complement to the standard parameter precision metrics.

Although it has been previously shown that the block-diagonal FIM may, in some examples, more accurately predict the empirical covariance matrix compared to the full FIM [10], this work has demonstrated that it may still be advantageous to use the full FIM for designs optimizations.

## Appendix

See Figs. 6, 7, 8, and 9.
